# Risk Factors for Sudden Infant Death in North Carolina

**DOI:** 10.3389/fped.2021.770803

**Published:** 2021-12-10

**Authors:** Merick M. Yamada, Michael B. Rosamilia, Karen E. Chiswell, Alfred D'Ottavio, Tracy Spears, Claire Osgood, Marie Lynn Miranda, Nina Forestieri, Jennifer S. Li, Andrew P. Landstrom

**Affiliations:** ^1^Department of Pediatrics, Division of Pediatric Cardiology, Duke University School of Medicine, Durham, NC, United States; ^2^Duke Clinical Research Institute, Duke University School of Medicine, Durham, NC, United States; ^3^Department of Applied and Computational Mathematics and Statistics, University of Notre Dame, Notre Dame, IL, United States; ^4^North Carolina Department of Health and Human Services, Raleigh, NC, United States; ^5^Department of Cell Biology, Duke University School of Medicine, Durham, NC, United States

**Keywords:** SIDS, SIDS (sudden infant death syndrome), race, ethnicity, infant mortality, birthweight, gestational age

## Abstract

**Background:** Sudden infant death syndrome (SIDS) is the sudden, unexplained death of infants <1 year old. SIDS remains a leading cause of death in US infants. We aim to identify associations between SIDS and race/ethnicity, birth weight/gestational age, and socioeconomic/environmental factors in North Carolina (NC) to help identify infants at risk for SIDS.

**Methods and Results:** In this IRB-approved study, infant mortality 2007–2016 and death certificate-linked natality 2007–2014 were obtained from the NC Department of Health and Human Services. General, NC natality statistics 2007–2016 were obtained from CDC Wonder. Association between SIDS/total infant death and covariates (below) were calculated. Total infant mortality decreased 2007–2016 by an average of 14 deaths/100,000 live births per year, while SIDS incidence remained constant. Risk ratios of SIDS/total infant deaths, standardized to Non-Hispanic White, were 1.76/2.41 for Non-Hispanic Black and 0.49/0.97 for Hispanic infants. Increased SIDS risk was significantly and independently associated with male infant sex, Non-Hispanic Black maternal race/ethnicity, young maternal age, low prenatal care, gestational age <39 weeks, birthweight <2500 g, low maternal education, and maternal tobacco use (*p* < *0.01*). Maternal previous children now deceased also trended toward association with increased SIDS risk.

**Conclusions:** A thorough SIDS risk assessment should include maternal, socioeconomic, and environmental risk factors as these are associated with SIDS in our population.

## Highlights

- Key Message: Sudden infant death syndrome remains a common cause of death in infants in North Carolina and has identifiable risk factors.
- What does it add to the existing literature: This study includes all SIDS cases in NC from 2007-2016, making it a larger and more contemporary population study.
- What is the impact: Low maternal education, low birth weight, less than 39 weeks gestational age, smoking, and non-Hispanic Black race are associated with SIDS. Hispanic ethnicity is a protective factor.

## Introduction

Sudden infant death syndrome, or SIDS, is the sudden unexplained death of an apparently healthy infant <1 year old with no clear cause of death after evaluation, traditional autopsy, and review of clinical history (1), and is a diagnosis of exclusion (2). SIDS remains one of the leading causes of death in infants in the United States (3, 4) with about 3,500 deaths annually (2). The pathophysiology of SIDS is thought to be multifactorial and governed by the “triple risk hypothesis”: an at-risk infant with underlying vulnerability that remains undefined, a critical development period in infancy, and exogenous risk factors (5). While recent evidence has suggested that only 10% of SIDS cases host likely pathogenic variants in channelopathy associated genes, this association represents an underlying susceptibility to cardiac arrhythmias in some SIDS infants (6–10). Further, there are known racial, socioeconomic, and environmental disparities in the incidence of SIDS (11), including Black race (2, 12), bed sharing, soft bedding, sleep surface, prematurity, smoking, birth weight, sleeping position, income, occupation, education, and housing (2, 13).

While total infant death has decreased across the United States, SIDS incidence has plateaued (4). This is true despite the national- and state-level interventions aimed at decreasing SIDS risk, including the passage of “The NC SIDS Law” in 2003 and the NC Back to Sleep Campaign. Given the persistence of SIDS deaths in the US, identification of additional risk factors associated with SIDS is needed to identify the at-risk infant. Previous studies have evaluated smaller groups of infants and have not studied multiple elements of the triple-risk hypothesis within the same cohort. In order to accurately evaluate SIDS risk in infants and direct preventative efforts accordingly, a recent, large sample, analyzing multiple elements of the triple risk hypothesis is necessary.

In this study, we evaluate a population-based cohort of deaths in North Carolina to identify associations between SIDS death and race/ethnicity, gestational age, birth weight, maternal level of education, tobacco use, and history of previous maternal child death (from any cause).

## Methods

### SIDS and Total Infant Mortality Data

This study was approved by the Duke University Health System Institutional Review Board. Infant mortality data were obtained from the North Carolina Department of Health and Human Services (NCDHHS). Data were abstracted from NC vital records from January 2007–July 2016. Data from 2016 were annualized in year-over-year analyses. The cohort of SIDS deaths had the following inclusion criteria: (1) NC death certificates from January 2007–July 2016, (2) age at death <1 year and not missing, and (3) underlying cause of death ICD10 code of R95 (“Sudden infant death syndrome”) or R99 (“Other ill-defined and unspecified causes of mortality”). Exclusion criteria included patients missing birth or death certificates. Death certificates from the latter part of 2016 were not received from NCDHHS and thus were not included in analyses. Demographic data from death records were identified using maternal, self-identified race and ethnicity. Birth certificate data from all NC births 2007–2014 were also obtained from NCDHHS. Birth certificates 2015–2016 were not included in analyses due to lack of availability of this data to investigators. Though the temporal range of death certificates included exceeded that of the birth certificates, only incidence by race/ethnicity over time was analyzed on death certificates. SIDS death records were linked with corresponding birth certificates by sex, date of birth, and full name. Partial matches, defined as any birth and death certificate with matching sex, date of birth, and a full name off by a single letter, were all included in the data set. Death records missing a matched birth certificate were excluded from analyses of covariates of SIDS risk.

### Natality Data

NC natality statistics were obtained from the CDC Wonder database 2007–2016 (14). Births were filtered by maternal bridged-race and maternal ethnicity with inclusion of Non-Hispanic White, Non-Hispanic Black, and Hispanic. CDC Wonder-derived birth data was used for analysis of demographic data to include the full cohort of SIDS death certificates 2007–2016.

### Analysis of Demographic Data

SIDS and total infant death incidences per 100,000 live births were calculated for race/ethnicity by year. As all data are from a complete population, inference testing was not performed. Change in incidence per year over the study period for SIDS and total infant death were calculated using a linear model. Mean differences between races/ethnicities by year over the study period for SIDS and total infant deaths, as well as related risk ratios were calculated. Risk ratios were standardized to Non-Hispanic White Incidence. Hispanic White and Hispanic Black are combined in the Hispanic group as >95% of all Hispanic births in the state of NC during the study period were Hispanic White.

### Analysis of Covariates of SIDS Risk

We selected potential covariates associated with SIDS risk from birth certificate data for analysis. This included sex of the baby, maternal race/ethnicity, maternal age at the time of birth (binned into 5-year intervals), Kotelchuck prenatal care index (categorized by sufficiency), obstetrician estimate of gestational age <39 weeks (compared with ≥39 weeks), birthweight <2500 g (compared with ≥2500 g), maternal education of 0–8 years or 9–12 years (compared with any college), any tobacco use during or in the year prior to pregnancy (compared with no tobacco use), and mother with at least one previous birth now dead (compared with mothers with no previous births now dead). Our cutoffs of gestational age, birthweight, and prenatal care are based on the typical designation of a full-term vs. preterm, low- vs. normal-birthweight infants, and sufficient prenatal care respectively (15–17). Divisions of tobacco smoking and maternal education variables were created based on logical cutoffs in our data set (ex. smoking vs. non-smoking).

The odds ratios of these covariates in SIDS infants vs. non-SIDS infants were calculated via univariate analysis and adjusted using a multivariable logistic regression model, including all mentioned covariates. All ORs were calculated for births 2007-−2014. All analyses were performed using RStudio V1.3 and SAS V9.4.

## Results

### Incidence of Sudden Infant Death Syndrome and Total Infant Death in North Carolina

**Figure 1:**
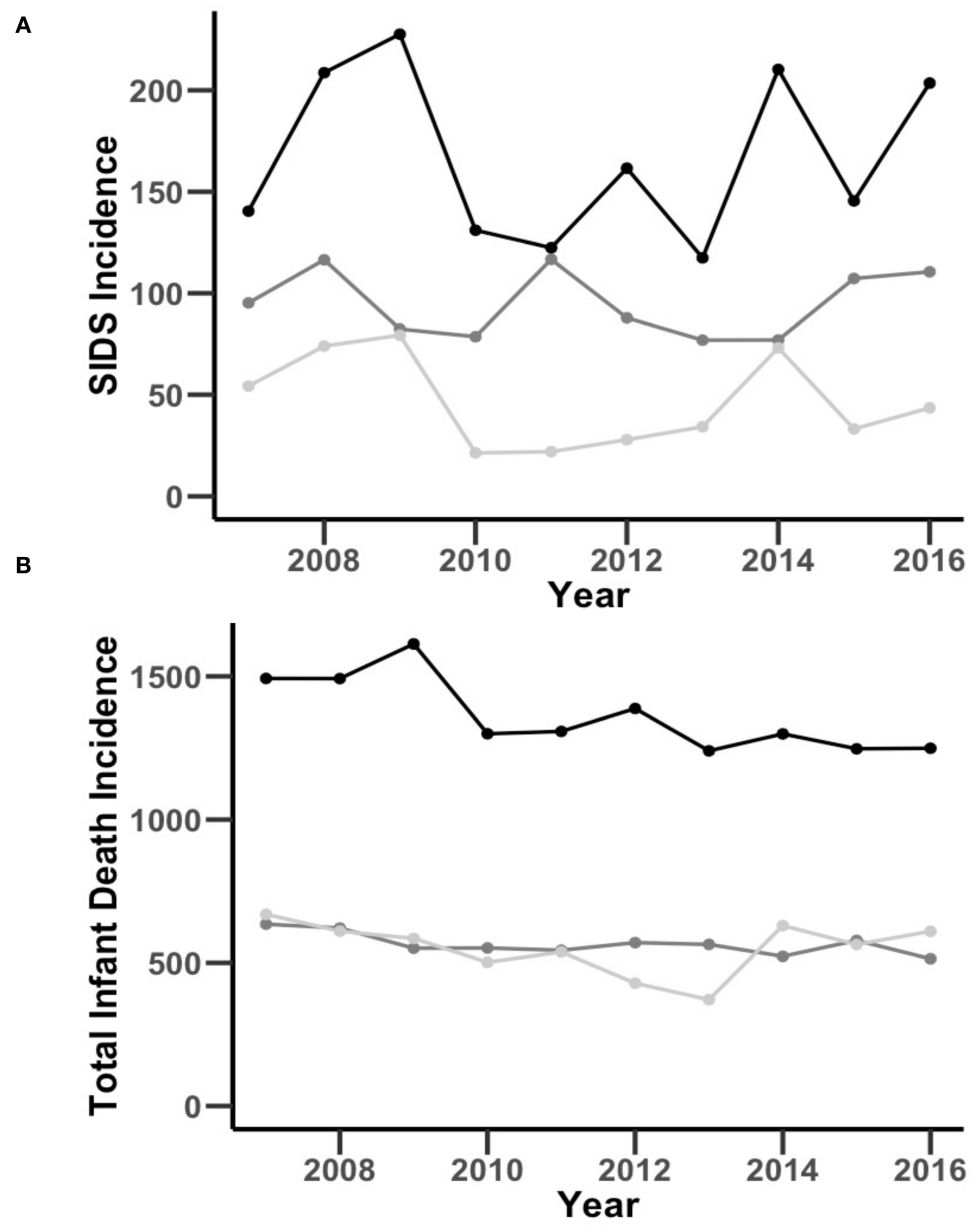
Comparison of NC SIDS **(A)** and total infant death **(B)** incidence per 100,000 live births by race and ethnicity over time through the years of 2007–2016. The black, dark gray, and light gray lines represent Non-Hispanic Black, Non-Hispanic White, and Hispanic incidence per 100,000 live births, respectively.

To determine the relationship between race/ethnicity and the incidence of SIDS, we analyzed NC death records and natality data 2007–2016. A total of 1,170,505 babies were born to mothers residing in the state of NC over the study period. There were 8,691 total infant deaths and 1,209 SIDS deaths, comprising 14% of total infant deaths (Table 1). A detailed flow diagram depicting the included and excluded cases is provided in Supplementary Figure 1. SIDS incidence over the study period was 105/100,000 live births with a non-significant change in incidence year-over-year (average decrease of 0.15 deaths/100,000 live births per year 95% CI [−4.3, 4.6]) (Figure 1). By race, SIDS incidence per 100,000 live births was 95 in Non-Hispanic White infants, 167 in Non-Hispanic Black infants, and 46 in Hispanic infants (Table 2). Non-Hispanic Black infants had a 1.76 risk ratio of SIDS compared to Non-Hispanic White infants. Interestingly, the Hispanic infants risk ratio of SIDS compared to Non-Hispanic White infants was 0.49 (Table 2). Overall, these findings show that the incidence of SIDS in NC has remained constant over the years 2007-2016 with persistent disparities between races/ethnicities disproportionately impacting Non-Hispanic Black infants. Further, Hispanic infants have a lower relative risk of SIDS compared to Non-Hispanic White infants.

**Table 1:** Demographics of the 2007–2016 cohort of SIDS, total infant deaths, and births.

	Total^*^	NH^**^White	**NH Black**	**Hispanic**
SIDS	1,155	607	462	86
Total Infant Death	8,546	3,713	3,831	1,002
Births	1,113,611	652,978	279,254	181,379

* *Total analyzed in the 2007–2016 cohort. Excludes other races (<5%) and missing data (<1%)*.

** *Non-Hispanic*.

**Table 2:** Relative risk of mortality incidence per 100,000 live births from SIDS and total infant death by race and ethnicity normalized to Non-Hispanic White.

	**Ethnicity**	**Risk Ratio**
SIDS	NH^*^ White	1
	NH Black	1.76
	Hispanic	0.49
Total	NH White	1
	NH Black	2.41
	Hispanic	0.97

* *Non-Hispanic*.

To determine the influence of race, ethnicity, and total infant death in SIDS, we first stratified population data by race and ethnicity. The overall incidence of total infant death was 763/100,000 live births with an average decrease of 14 deaths/100,000 live births per year (95% CI [−22, −6.0], Figure 1). Total infant death incidence per 100,000 live births was 565 in Non-Hispanic White infants, 1,363 in Non-Hispanic Black infants, and 551 in Hispanic infants. Non-Hispanic Black infants had a 2.41 risk ratio and Hispanic infants had a 0.97 risk ratio of infant mortality compared to Non-Hispanic Caucasian infants (Table 2). Together, these findings show that there was a decrease in the incidence of total infant death in NC from 2007-2016, but a persistent, disproportionate impact on Non-Hispanic Blacks in both SIDS and total infant death.

### Associations With Sudden Infant Death Syndrome

**Figure 2:**
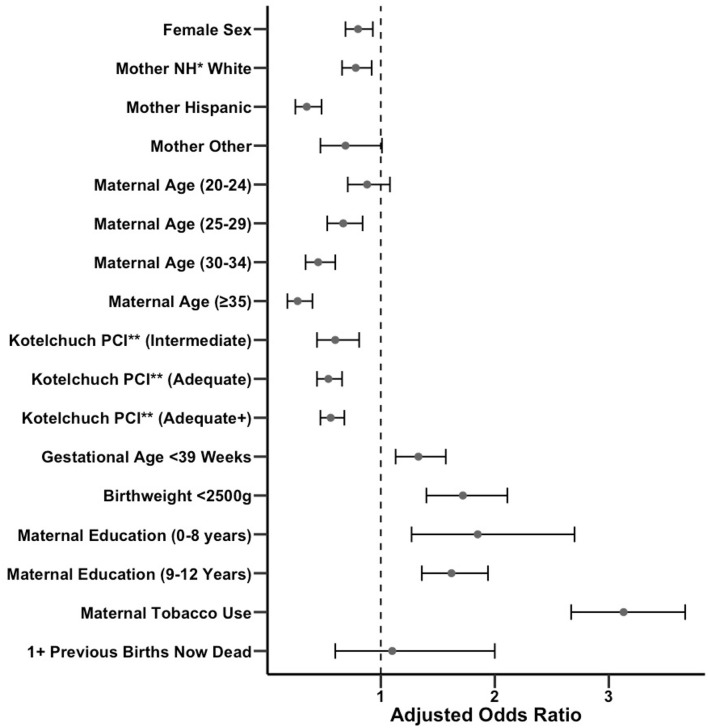
Forest plot of adjusted odds ratios for exposure to various covariates in SIDS vs. non-SIDS infants with confidence intervals. Unadjusted odds ratios are also provided in Supplementary Table 2. *NH, Non-Hispanic. **PCI, Prenatal Care Index.

To determine the relationship between SIDS incidence and covariates of sex of the baby, maternal race/ethnicity, maternal age at the time of birth, Kotelchuck prenatal care index, gestational age, birthweight, maternal education, tobacco use, and mother with at least one previous birth now dead, we performed a multivariable logistic regression comparing births resulting in a SIDS death vs. all other births, yielding adjusted odds ratios (ORs). A table of SIDS and non-SIDS infant proportions by covariate is included in Supplemental Table 1. The odds of developing SIDS was significantly (*p* < *0.01*) decreased with female sex (OR 0.80), non-Hispanic White or Hispanic race/ethnicity compared with non-Hispanic Black (OR 0.78 and 0.35, respectively), maternal age of 25–29, 30–34, and ≥35 compared with ≤19 (ORs 0.67, 0.45, and 0.27, respectively), Kotelchuck prenatal care index of intermediate, adequate, and adequate plus sufficiency (ORs 0.60, 0.54, and 0.56, respectively), and increased with gestational age <39 weeks (OR 1.33), birthweight <2500 g (OR 1.72), maternal education of 0–8 years or 9–12 years (ORs 1.85 and 1.62, respectively), and any tobacco use during or in the three months prior to pregnancy relative to non-SIDS infants (OR 3.13). The odds of developing SIDS trended toward a decrease with maternal age 20–24 years compared with ≤19 years (OR 0.88, *p* = *0.23*), or other race/ethnicity compared with Non-Hispanic Black (OR 0.69, *p* = *0.056*), and an increase when infant's mother had at least one previous birth now dead (OR 1.10, *p* = *0.76*). All adjusted ORs are summarized in Figure 2. We also calculated univariate associations with SIDS incidence (Supplementary Table 2). Taken together, these findings suggest that numerous host and environmental factors are significantly associated with SIDS incidence.

## Discussion

In 1992, the American Academy of Pediatrics debuted the Back to Sleep Campaign recommending that babies be put to sleep in the non-prone position, which led to a decrease in total SIDS deaths by 40%. Unfortunately, in the past two decades, there has been a stable number of SIDS deaths in the United States (2). Concordant with national statistics, we observed that the total incidence of infant death in NC has decreased, but SIDS incidence has remained constant. Our results also agree with other studies that have shown that Non-Hispanic Black infants are at a disproportionately high SIDS risk (12, 18). We also find that lower maternal education level (19), lower maternal age, male sex of the baby, and prior sibling death are associated with increased SIDS risk (20). Many of these associations have been previously shown in other studies; however, this study utilizes a more robust statistical approach, with a multivariable model, in a larger and more recent population to demonstrate independent risk factors for SIDS. Of note, incidence of SIDS and total infant death was higher in NC compared with all US births (105/100,000 vs. 73/100,000 live births for SIDS and 763/100,000 vs. 560/100,000 live births for total infant death respectively). Furthermore, NC rates of SIDS risk factors exceeded statistics for the entire US population including a higher smoking rate before/during pregnancy (12.2 vs. 9.4%) and a lower average birthweight (3,258 grams vs. ~3,500 grams) (21, 22). Overall, our data are in agreement with prior studies about SIDS risk factors, with novel elements including a state-wide population analysis with a larger sample size, a more recent cohort, and a larger number of studied covariates.

Other studies have attempted to explain the racial disparity in SIDS. For example, it has been described that racial discrimination affecting Black women is a risk factor for poor pregnancy outcomes, which seems to be related to chronic stress leading to enhanced inflammatory response, compromised fetal development and adverse pregnancy outcomes (12, 23). Examples of adverse pregnancy outcomes include low birthweight and prematurity which, as we have described, are both correlated to SIDS risk. Therefore, there is a highly plausible connection between racial disparities and SIDS, mediated, in part, by pregnancy outcomes. Additionally, there are cultural factors that may play a role in infant care practices (24). As has been shown in previous studies (25, 26), we found that Hispanic ethnicity was associated with a reduction in SIDS incidence. Previous literature has suggested protective factors that are more prevalent in Hispanic families that may lead to a lower SIDS risk, including multigenerational home and greater family support. In summary, the racial and ethnic disparities we observed are potentially explained by outcomes related to systemic racism and cultural differences.

While we did not directly examine the pathways underlying biological or environmental associations with SIDS, we can generate hypotheses for explanatory mechanisms for directly analyzed covariates based on previous literature. Explanations for the association between prematurity and/or low birthweight and SIDS relate to problems of underdevelopment of respiratory control. For example, a 1998 study proposed a link between lung underdevelopment, known as apnea of prematurity, and SIDS (27). Furthermore, underdeveloped respiratory centers in premature and low birthweight babies may play a role in elevating SIDS risk (28). There are also several likely contributors in the causal pathway between maternal smoking and SIDS, including elevated respiratory infection risk and worse pregnancy outcomes, such as prematurity and low birthweight (29–31). Regarding the observed association between maternal education and SIDS, there are clear links between adequacy of pre-natal care, education level and health literacy (32). Given that most SIDS prevention efforts to date have focused on parental education and behavior modification for infant care, a link between health literacy and SIDS is quite likely. Of note, regarding prenatal care specifically, we found that the majority of SIDS risk reduction from increasing prenatal care came from moving from the inadequate prenatal care group to the intermediate prenatal care group (17). This finding reinforces the goal of maximizing prenatal care, even when “adequate” levels cannot be reached. Finally, while not statistically significant, we observed an association between SIDS and at least one of the mother's previous births now deceased. This association can be explained both by the growing literature on evidence of the role of genetic predisposition in SIDS, as well as the fact that siblings may experience similar parental child-care practices (20, 33, 34). To gain more insight into the heritability of SIDS on a population level, sibling and twin studies must be performed. Overall, there is at least one plausible explanation linking each of our observed risk factors to SIDS.

One potential factor influencing SIDS risk that we did not directly measure due to data constraint is insurance status, as this influences access to healthcare and health education resources and has been associated with many health outcomes. Mothers in NC, along with newborn infants, have a mix of insurance types, private and public. In NC, all infants born to mothers with Medicaid are automatically guaranteed enrollment in Medicaid until they turn 1 year of age. Medicaid is also available to qualifying mothers while pregnant and for 60 days post-partum (35). Of note, in SIDS, insurance status has not been shown to be an independent risk factor in models incorporating income (36).

As we have observed, SIDS is multifactorial and complex. Improved understanding of risk factors and health disparities can improve our care for these populations and, hopefully, lead to a decrease in SIDS.

### Limitations

This study is retrospective and observational in nature. Additionally, not all birth certificates were available for the entire SIDS death population, and birth certificates were not available to us after 2014, meaning that the cohort for racial/ethnic associations was somewhat different from that involved in calculation of other variable associations.

## Conclusions

In our study of NC deaths 2007–2016, we find that the incidence of total infant death has decreased over time while SIDS incidence has remained constant, with a higher SIDS risk in Non-Hispanic Black infants, and a lower SIDS risk in Hispanic infants compared to Non-Hispanic White infants. Additional observed variables associated with SIDS include male sex, low prenatal care, prematurity, low birthweight, low maternal education, maternal smoking, and having a previous child die. The considerable differences in SIDS incidence related to biological and environmental conditions highlight the need for SIDS risk assessment, along with a concentration of effort into reducing health disparities in pregnancy and early life.

## Data Availability Statement

The raw data supporting the conclusions of this article will be made available by the authors, without undue reservation.

## Ethics Statement

The studies involving human participants were reviewed and approved by Duke University Health System Institutional Review Board. Written informed consent for participation was not required for this study in accordance with the national legislation and the institutional requirements. Written informed consent was not obtained from the individual(s) for the publication of any potentially identifiable images or data included in this article.

## Disclosure

This manuscript was previously presented as a poster at the Duke University School of Medicine Department of Pediatrics Retreat and the American Academy of Pediatrics Conference 2021.

## Author Contributions

MY, MR, CO, MM, JL, and AL: planned the study, interpreted the findings, drafted the manuscript and/or revised the manuscript. NF: provided data. KC, AD'O, and TS: provided data support, analysis, and statistical analysis. JL and AL: provided study oversight. All authors contributed to the article and approved the submitted version.

## Funding

This study received funding from Pfizer Foundation grant (MR training fellowship) and the Duke Clinical Translational Science Institute (MR training fellowship). These funders were not involved in the study design, collection, analysis, interpretation of data, and the writing of the article or the decision to submit for publications. This study also received funding from the Centers for Disease Control and Prevention (NU50DD004933-03-00). JL is supported by grants from NCATS, CDC, and AHA. AL is supported by grants from the NIH, CDC, American Sudden Infant Death Syndrome Institute, and the Duke Children's Health and Discovery Initiative.

## Conflict of Interest

MR received a consulting fee from Nabla Bio for an unrelated project. The remaining authors declare that the research was conducted in the absence of any commercial or financial relationships that could be construed as a potential conflict of interest.

## Publisher's Note

All claims expressed in this article are solely those of the authors and do not necessarily represent those of their affiliated organizations, or those of the publisher, the editors and the reviewers. Any product that may be evaluated in this article, or claim that may be made by its manufacturer, is not guaranteed or endorsed by the publisher.

## Abbreviations

SIDS: sudden infant death syndrome
NC: North Carolina
CDC: Centers for Disease Control.
