# Supplementary material for: Risk Factors for Sudden Infant Death in North Carolina
Source: Front Pediatr. 2021 Dec 10;9:770803. doi: 10.3389/fped.2021.770803 (PMC8703192; doi:10.3389/fped.2021.770803)
Supplement: Supplementary file 1 [file Data_Sheet_1.pdf]

Risk Factors for Sudden Infant Death in North Carolina

Supplemental Materials

Yamada *et al*.

Supplemental Results

Supplemental Figure 1: Flow diagram depicting included and excluded cases in all analyses, along with rationales for exclusion.

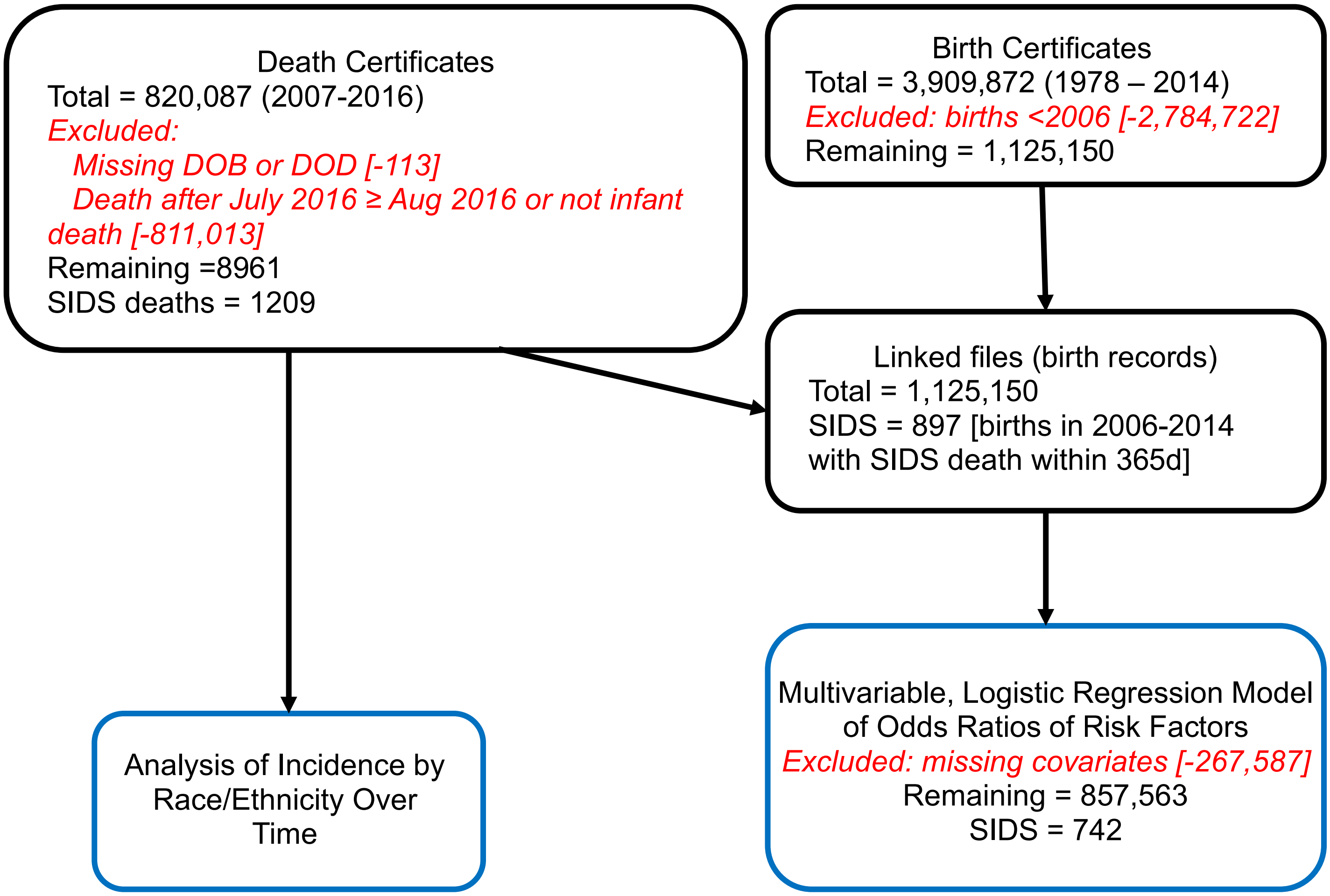

Supplemental Table 1: Number and proportions associated with all covariates used in the multivariable logistic regression model of SIDS risk factors in both SIDS and All NC cohorts 2007-2014. Additionally, median and IQR are included for certain variables. Data for mother education level, tobacco use, and Kotelchuck Prenatal Care Index was not received for 2010 and is thus excluded here.

	SIDS (N=897)	All NC (N=1,125,150)
**Sex, n (%)**		
Male	507 (56.5)	575,474 (51.1)
Female	390 (43.5)	549,661 (48.9)
Unknown	0 (0.0)	15 (0.0)
**Mother Age (yrs)**		
Median (25th, 75th)	23.0 (21.0, 27.0)	27.0 (22.0, 32.0)
**Mother Age, n (%)**		
≤19	156 (17.4)	110,863 (9.9)
20-24	366 (40.8)	291,512 (25.9)
25-29	232 (25.9)	312,624 (27.8)
30-34	101 (11.3)	260,423 (23.1)
35-39	34 (3.8)	123,042 (10.9)
≥40	8 (0.9)	26,610 (2.4)
**Mother Race/Ethnicity, n (%)**		
Non-Hispanic Black	325 (36.2)	269,177 (23.9)
Hispanic	54 (6.0)	172,369 (15.3)
Non-Hispanic White	486 (54.2)	627,799 (55.8)
Other	32 (3.6)	55,805 (5.0)
**Previous births now dead, n (%)**		
None	881 (98.2)	1,111,404 (98.8)
One	15 (1.7)	11,903 (1.1)
Two	1 (0.1)	1,073 (0.1)
>Two	0 (0.0)	281 (0.0)
Unknown	0 (0.0)	489 (0.0)
**Obstetric estimate of gestation (weeks)**		
Median (25th, 75th)	39.0 (37.0, 39.0)	39.0 (38.0, 40.0)
**Birthweight (grams)**		
Median (25th, 75th)	3033.5 (2614.1, 3374.0)	3317.0 (2948.4, 3630.0)
**Education of Mother, n (%)**		
0-8 years of Primary/Secondary school	40 (5.0)	57,721 (5.8)
9-12 years of Primary/Secondary school	500 (62.8)	396,903 (39.6)
Any college	256 (32.2)	545,571 (54.4)
Unknown	0 (0.0)	1,916 (0.2)

**Tobacco Use, n (%)**		
**Yes**	**311 (39.1)**	**122,288 (12.2)**
**No**	**485 (60.9)**	**879,043 (87.7)**
**Unknown**	**0 (0.0)**	**780 (0.1)**
**Kotelchuck Prenatal Care Index, n (%)**		
**Missing Information**	**29 (3.6)**	**15,838 (1.6)**
**Inadequate**	**218 (27.4)**	**132,593 (13.2)**
**Intermediate**	**53 (6.7)**	**80,764 (8.1)**
**Adequate**	**203 (25.5)**	**351,771 (35.1)**
**Adequate Plus**	**293 (36.8)**	**421,145 (42.0)**

Supplemental Table 2: Odds ratios for the SIDS cohort compared with all NC births for each covariate of interest by level. Reference levels are indicated. Graphical representation of these odds ratios with 95% CIs are depicted in Figure 2 of the primary article.

Covariate	Odds Ratio (95% CI)	p-Value
**Female Sex**		
No	Reference	
Yes	0.80 (0.70 - 0.92)	0.001
**Maternal Age (Years)**		
≤19	Reference	
20-24	0.86 (0.71 - 1.04)	0.125
25-29	0.51 (0.41 - 0.62)	<.001
30-34	0.26 (0.20 - 0.34)	<.001
≥35	0.19 (0.13 - 0.26)	<.001
**Mother Race/Ethnicity**		
Black	Reference	
Hispanic Non-Black	0.26 (0.20 - 0.35)	<.001
White	0.46 (0.32 - 0.67)	<.001
Other	0.63 (0.55 - 0.73)	<.001
**Kotelchuck Prenatal Care Index (PCI)**		
Inadequate	Reference	
Intermediate	0.35 (0.29 - 0.43)	<.001
Adequate	0.43 (0.36 - 0.52)	<.001
Adequate Plus	0.43 (0.31 - 0.57)	<.001
**Previous Births Now Dead**		
None	Reference	
1 or more	1.61 (0.98 - 2.64)	0.059
**Obstetric Estimate of Gestational Age**		
≥39 weeks	Reference	
<39 weeks	1.71 (1.49 - 1.95)	<.001
**Birthweight**		
≥2500 g	Reference	
<2500 g	2.68 (2.28 - 3.15)	<.001
**Education of Mother**		
Any college	Reference	
0-8 years of primary/secondary	1.58 (1.13 - 2.21)	0.008
9-12 years of primary/secondary	2.80 (2.41 - 3.27)	<.001
**Tobacco Use**		
No	Reference	
Yes	4.68 (4.05 - 5.41)	<.001
